# Supplementary material for: Haitian coffee agroforestry systems harbor complex arabica variety mixtures and under-recognized genetic diversity
Source: PLoS One. 2024 Apr 16;19(4):e0299493. doi: 10.1371/journal.pone.0299493 (PMC11020479; doi:10.1371/journal.pone.0299493)
Supplement: S1 Table — (DOCX) [file pone.0299493.s001.docx]

Supplementary data

Haitian coffee agroforestry systems harbor complex Arabica variety mixtures and under-recognized genetic diversity.

Claude Patrick Millet (claudepatrickmillet@gmail.com), Clémentine Allinne, Tram Vi, Pierre Marraccini, Lauren Verleysen, Marie Couderc, Tom Ruttink, Dapeng Zhang, William Solano-Sanchéz, Christine Tranchant-Dubreuil, Wesly Jeune, Valérie Poncet

**Table S1. Name, location and number of samples for each study site**. The municipality Grande-Rivière-du-Nord is abbreviated as GRN.

| **Farm ID** | **Department** | **Municipality** | **Locality name** | **Latitude** | **Longitude** | **Number of plants sampled** |
| --- | --- | --- | --- | --- | --- | --- |
| **G05** | Grande-Anse | Beaumont | Belance | 18.4672105 | -73.97903 | 24 |
| **G06** | Grande-Anse | Beaumont | Bois Misquette | 18.4763379 | -73.976884 | 20 |
| **G07** | Grande-Anse | Beaumont | Bois Lacombe | 18.4490638 | -73.976122 | 22 |
| **G08** | Grande-Anse | Beaumont | Fonds Cochon | 18.4883196 | -73.97104 | 20 |
| **G09** | Grande-Anse | Beaumont | Bois Pin | 18.4485387 | -73.962085 | 22 |
| **G10** | Grande-Anse | Beaumont | Labondance | 18.4749466 | -73.943939 | 20 |
| **G11** | Grande-Anse | Beaumont | Savane Zidor | 18.4821107 | -73.920777 | 20 |
| **G12** | Grande-Anse | Pestel | Tozia | 18.451436 | -73.868033 | 26 |
| **G13** | Grande-Anse | Pestel | K-Gous | 18.5289797 | -73.828653 | 20 |
| **G14** | Grande-Anse | Pestel | Mabilai | 18.4987137 | -73.811 | 23 |
| **G15** | Grande-Anse | Pestel | Desriveaux | 18.4713702 | -73.810397 | 21 |
| **G16** | Grande-Anse | Pestel | Billard Dépôt | 18.490385 | -73.801059 | 20 |
| **G17** | Grande-Anse | Pestel | Jeanbellune | 18.480481 | -73.783794 | 21 |
| **G18** | Grande-Anse | Pestel | Mentor | 18.4723964 | -73.77081 | 22 |
| **N01** | Nord | Dondon | Grand Chemin | 19.5459768 | -72.269341 | 25 |
| **N02** | Nord | Dondon | Fleury | 19.4915679 | -72.261885 | 24 |
| **N03** | Nord | Dondon | Clément | 19.5487275 | -72.258205 | 24 |
| **N04** | Nord | Dondon | Matador | 19.5037206 | -72.254102 | 26 |
| **N05** | Nord | Dondon | Bernice | 19.5224983 | -72.248129 | 25 |
| **N06** | Nord | Dondon | Bellevue | 19.5123115 | -72.243898 | 24 |
| **N07** | Nord | Dondon | Lanneau | 19.5339873 | -72.23641 | 24 |
| **N08** | Nord | Dondon | Michel | 19.5520965 | -72.235948 | 24 |
| **N09** | Nord | Dondon | Bois Rouge | 19.542891 | -72.224254 | 23 |
| **N10** | Nord | GRN | Vieux Cayes | 19.5542768 | -72.198133 | 21 |
| **N11** | Nord | Baron | Muscady | 19.4739142 | -72.11539 | 20 |
| **N12** | Nord | Baron | Cadette | 19.4748559 | -72.085731 | 9 |
| **N13** | Nord | Baron | Mompoint | 19.4687234 | -72.084296 | 20 |
| **N14** | Nord | Baron | Grenier | 19.4778762 | -72.08105 | 20 |
