# Supplementary material for: Haitian coffee agroforestry systems harbor complex arabica variety mixtures and under-recognized genetic diversity
Source: PLoS One. 2024 Apr 16;19(4):e0299493. doi: 10.1371/journal.pone.0299493 (PMC11020479; doi:10.1371/journal.pone.0299493)
Supplement: S2 Table — List of reference samples by code and accession name, country and region of origin (when this information is known to the authors), and name of the holding institution whose collection the sample was acquired from. (DOCX) [file pone.0299493.s002.docx]

**Table S2**. **List of sample accessions used as references in the study**. List of reference samples by code and accession name, country and region of origin (when this information is known to the authors), and name of the holding institution whose collection the sample was acquired from. Also mentioned is whether the accession was used in the study that selected the core SNP panel used in the KASP SNP genotyping assay (Zhang et al., 2021), and what genetic group the sample was assigned to with sNMF population structure analysis using KASP SNP data (at K=6) and HiPlex haplotype data (at K=7). Genetic groups are abbreviated as follows: Typ = Typica-like, CR = CR95 (Catimor)-like, BBN = Bourbon-like, Kent = Kent/I-60-like, Ethio = Ethiopian-like, Admix = admixed assignment. “N/A” in either column indicates that the sample was not represented in the corresponding dataset.

| **Sample**  **Code** | **Accession** | **Country of origin** | **Region of origin** | **used by Zhang et al., 2021** | **Holding institution** | **Assign KASP K=6** | **Assign HiPlex**  **K=7** |
| --- | --- | --- | --- | --- | --- | --- | --- |
| **AR 56_5** | *C. arabica* | Ethiopia |  |  | IRD, France | Ethio | HiPlex6 |
| **AR15_5** | *C. arabica* | Ethiopia |  |  | IRD, France | Ethio | Admix |
| **AR22_5** | *C. arabica* | Ethiopia |  |  | IRD, France | Ethio | HiPlex6 |
| **AR41_6** | *C. arabica* | Ethiopia |  |  | IRD, France | Ethio | HiPlex6 |
| **BA58** | *C. canephora* | Ivory Coast |  |  | IRD, France | N/A | N/A |
| **BB53** | *C. canephora* | CAR |  |  | IRD, France | N/A | N/A |
| **CA54** | *C. congensis* | CAR |  |  | IRD, France | N/A | N/A |
| **CC53** | *C. congensis* | Congo |  |  | IRD, France | N/A | N/A |
| **CTR** | Caturra | CATIE collection |  |  | IRD, France | Admix | Admix |
| **EA64** | *C. liberica var. liberica* | Ivory Coast |  |  | IRD, France | N/A | Admix |
| **EB52** | *C. liberica var. dewevrei* | CAR |  |  | IRD, France | N/A | N/A |
| **EB58** | *C. liberica var. dewevrei* | CAR |  |  | IRD, France | N/A | N/A |
| **GUI2** | *C. canephora* | Guinea |  |  | IRD, France | N/A | N/A |
| **HA_13** | *C. arabica* |  |  |  | IRD, France | N/A | N/A |
| **T.00977** | Blue Mountain | Guatemala | La Aurora | Yes | CATIE, Costa-Rica | Typ | N/A |
| **T.00989** | Guadeloupe | El Salvador | La Libertad | Yes | CATIE, Costa-Rica | Typ | HiPlex1 |
| **T.00990** | Surinam | El Salvador | La Libertad | Yes | CATIE, Costa-Rica | Typ | HiPlex1 |
| **T.01993** | Goiaba | Brasil | Campinas | Yes | CATIE, Costa-Rica | Kent | HiPlex1 |
| **T.02147** | Murta | Guatemala | La Aurora | Yes | CATIE, Costa-Rica | BBN | Admix |
| **T.02246** | Jimma-1 | Ethiopia | Jimma | Yes | CATIE, Costa-Rica | Ethio | HiPlex5 |
| **T.02249** | Dessie | Ethiopia | Dessie | Yes | CATIE, Costa-Rica | Admix | HiPlex6 |
| **T.02251** | Batie | Ethiopia | Dessie | Yes | CATIE, Costa-Rica | Admix | Admix |
| **T.02254** | Jimma-6 | Ethiopia | Jimma | Yes | CATIE, Costa-Rica | Ethio | Admix |
| **T.02257** | Lekemti | Ethiopia | Lekemti | Yes | CATIE, Costa-Rica | Admix | HiPlex6 |
| **T.02298** | Coorg | Kenya | Ruiru | Yes | CATIE, Costa-Rica | Typ | Admix |
| **T.02299** | Laurina | Costa Rica | Cartago | Yes | CATIE, Costa-Rica | Admix | Admix |
| **T.02394** | Mocha Java | Puerto Rico | Mayaguez | Yes | CATIE, Costa-Rica | BBN | Admix |
| **T.02395** | Erecta | Puerto Rico | Mayaguez | Yes | CATIE, Costa-Rica | Typ | Admix |
| **T.02542** | Caturra | Brasil | Campinas | Yes | CATIE, Costa-Rica | BBN | Admix |
| **T.02676** | Laurina | Camerun | Dschang | Yes | CATIE, Costa-Rica | Admix | Admix |
| **T.02702** | Mibirizi | Congo | N/A | Yes | CATIE, Costa-Rica | Admix | Admix |
| **T.02727** | Dalle | Kenya | Sidamo | Yes | CATIE, Costa-Rica | Ethio | Admix |
| **T.02731** | Jimma Galla Sidamo | Kenya | N/A | Yes | CATIE, Costa-Rica | Ethio | Admix |
| **T.02741** | Erecta | Kenya | N/A | Yes | CATIE, Costa-Rica | BBN | HiPlex6 |
| **T.02742** | Dilla Alghe | Ethiopia | Sidamo | Yes | CATIE, Costa-Rica | Admix | HiPlex5 |
| **T.02744** | Rume Sudan | Kenya | N/A | Yes | CATIE, Costa-Rica | Admix | Admix |
| **T.02758** | Barbuk Sudan | Sudan | Barbuk | Yes | CATIE, Costa-Rica | Admix | N/A |
| **T.03081** | Carrizal | Costa Rica | Alajuela | Yes | CATIE, Costa-Rica | Admix | Admix |
| **T.03214** | Geisha | Tanzania | N/A | Yes | CATIE, Costa-Rica | Admix | Admix |
| **T.03215** | K-7 | Kenya | N/A | Yes | CATIE, Costa-Rica | Admix | Admix |
| **T.03427** | Cera | Brasil | Campinas | Yes | CATIE, Costa-Rica | Typ | Admix |
| **T.03443** | Ceilan | Puerto Rico | Mayaguez | Yes | CATIE, Costa-Rica | Typ | HiPlex1 |
| **T.03469** | Bourbon Salvadoreno | El Salvador | La Libertad | Yes | CATIE, Costa-Rica | BBN | HiPlex1 |
| **T.03491** | Lejeune 08 | Ethiopia | Bada Buna | Yes | CATIE, Costa-Rica | Ethio | Admix |
| **T.03507** | Lejeune 12 | Ethiopia | Bada Buna | Yes | CATIE, Costa-Rica | Ethio | HiPlex6 |
| **T.03645** | Cumbaya |  |  |  | CATIE, Costa-Rica | Typ | HiPlex6 |
| **T.04007** | Loulo | Ethiopia | Sidamo | Yes | CATIE, Costa-Rica | Admix | N/A |
| **T.04060** | Murta | Isla Reunión | N/A | Yes | CATIE, Costa-Rica | Admix | Admix |
| **T.04076** | Typica Amarillo | Colombia | Caldas | Yes | CATIE, Costa-Rica | Admix | Admix |
| **T.04078** | Caturra Variegata | Colombia | Caldas | Yes | CATIE, Costa-Rica | BBN | Admix |
| **T.04250** | Goiaba | Colombia | Caldas | Yes | CATIE, Costa-Rica | Admix | HiPlex5 |
| **T.04253** | Maragogipe | Colombia | Caldas | Yes | CATIE, Costa-Rica | Admix | Admix |
| **T.04258** | Bourbon Mayaguez | Congo | Ruanda Urundi | Yes | CATIE, Costa-Rica | Admix | Admix |
| **T.04259** | Jackson 2 | Congo | Ruanda Urundi | Yes | CATIE, Costa-Rica | Admix | Admix |
| **T.04268** | Kent | Congo | Ruanda Urundi | Yes | CATIE, Costa-Rica | Kent | Admix |
| **T.04271** | Kent | Congo | Ruanda Urundi | Yes | CATIE, Costa-Rica | Admix | Admix |
| **T.04273** | Kabare | Congo | Ruanda Urundi | Yes | CATIE, Costa-Rica | Admix | Admix |
| **T.04278** | Babaca Kaffa | Congo | Ruanda Urundi | Yes | CATIE, Costa-Rica | Admix | Admix |
| **T.04281** | Lignee-M | Congo | Ruanda Urundi | Yes | CATIE, Costa-Rica | Admix | Admix |
| **T.04286** | Wondo Sidamo | Congo | Ruanda Urundi | Yes | CATIE, Costa-Rica | Ethio | Admix |
| **T.04290** | Jimma Kaﬀa | Congo | Ruanda Urundi | Yes | CATIE, Costa-Rica | Ethio | Admix |
| **T.04292** | Wush Wush Kaﬀa | Congo | Ruanda Urundi | Yes | CATIE, Costa-Rica | Ethio | HiPlex6 |
| **T.04294** | Mocha de Tahiti | Congo | Ruanda Urundi | Yes | CATIE, Costa-Rica | Typ | HiPlex6 |
| **T.04308** | Dalle mixed | Malawi | N/A | Yes | CATIE, Costa-Rica | Ethio | HiPlex1 |
| **T.04310** | Mocha | Malawi | N/A | Yes | CATIE, Costa-Rica | Admix | Admix |
| **T.04313** | SL28 |  |  |  | CATIE, Costa-Rica | Admix | Admix |
| **T.04314** | SL34 |  |  |  | CATIE, Costa-Rica | Admix | Admix |
| **T.04317** | I-60 | Malawi | N/A | Yes | CATIE, Costa-Rica | Kent | Admix |
| **T.04375** | Bourbon Amarillo | Venezuela | Monajas | Yes | CATIE, Costa-Rica | Admix | Admix |
| **T.04479** | E-301 | Ethiopia | Kaﬀa Jimma | Yes | CATIE, Costa-Rica | Admix | N/A |
| **T.04539** | E-293 | Ethiopia | Kaﬀa Jimma | Yes | CATIE, Costa-Rica | Ethio | N/A |
| **T.04570** | E-147 | Ethiopia | Illubabor | Yes | CATIE, Costa-Rica | Ethio | N/A |
| **T.04573** | E-150 | Ethiopia | Illubabor | Yes | CATIE, Costa-Rica | Ethio | Admix |
| **T.04578** | E-155 | Ethiopia | Illubabor | Yes | CATIE, Costa-Rica | Ethio | HiPlex6 |
| **T.04583** | E-325 | Ethiopia | Illubabor | Yes | CATIE, Costa-Rica | Ethio | HiPlex6 |
| **T.04599** | E-341 | Ethiopia | Kaﬀa Jimma | Yes | CATIE, Costa-Rica | Ethio | Admix |
| **T.04610** | E-352 | Ethiopia | Kaﬀa Jimma | Yes | CATIE, Costa-Rica | Ethio | Admix |
| **T.04667** | E-160 | Ethiopia | Kaﬀa Jimma | Yes | CATIE, Costa-Rica | Ethio | HiPlex6 |
| **T.04681** | E-167 | Ethiopia | Kaﬀa Jimma | Yes | CATIE, Costa-Rica | Ethio | N/A |
| **T.04688** | E-174 | Ethiopia | Kaﬀa Jimma | Yes | CATIE, Costa-Rica | Ethio | HiPlex6 |
| **T.04692** | E-178 | Ethiopia | Kaﬀa Jimma | Yes | CATIE, Costa-Rica | Ethio | N/A |
| **T.05175** | IHCAFE 90 |  |  |  | CATIE, Costa-Rica | Admix | HiPlex6 |
| **T.05199** | Cioiccie I | Colombia | Caldas | Yes | CATIE, Costa-Rica | Admix | Admix |
| **T.05267** | Catuai | Costa Rica | San José | Yes | CATIE, Costa-Rica | Admix | Admix |
| **T.05268** | Catuai | Costa Rica | San José | Yes | CATIE, Costa-Rica | Admix | Admix |
| **T.05283** | Cioiccie S-6 | Brasil | Campinas | Yes | CATIE, Costa-Rica | Admix | Admix |
| **T.05314** | Hibrido_041 |  |  |  | CATIE, Costa-Rica | Admix | HiPlex6 |
| **T.05325** | Catuai | Costa Rica | Alajuela | Yes | CATIE, Costa-Rica | Admix | Admix |
| **T.08867** | CR 95 |  |  |  | CATIE, Costa-Rica | CR | Admix |
| **T.11723** | Garnica | Mexico | Veracruz | Yes | CATIE, Costa-Rica | BBN | N/A |
| **T.11948** | Hibrido_101 |  |  |  | CATIE, Costa-Rica | Admix | Admix |
| **T.11950** | Clon 7355 | Ethiopia | N/A | Yes | CATIE, Costa-Rica | Admix | Admix |
| **T.12534** | Clon 7357 | Ethiopia | N/A | Yes | CATIE, Costa-Rica | Typ | Admix |
| **T.12837** | Hibrido_055 | Mexico | Veracruz | Yes | CATIE, Costa-Rica | Admix | Admix |
| **T.12841** | Catimor | Mexico | Veracruz | Yes | CATIE, Costa-Rica | Admix | Admix |
| **T.12842** | Catimor | Mexico | Veracruz | Yes | CATIE, Costa-Rica | Admix | Admix |
| **T.12846** | Hibrido_089 | Mexico | Veracruz | Yes | CATIE, Costa-Rica | Ethio | Admix |
| **T.12851** | Garnica | Mexico | Veracruz | Yes | CATIE, Costa-Rica | Admix | Admix |
| **T.14718** | Sarchimor |  |  |  | CATIE, Costa-Rica | Admix | Admix |
| **T.14723** | Icatu | Brasil | Campinas | Yes | CATIE, Costa-Rica | Admix | Admix |
| **T.14724** | Catimor |  |  |  | CATIE, Costa-Rica | Admix | Admix |
| **T.15895** | Pacas | El Salvador | La Libertad | Yes | CATIE, Costa-Rica | BBN | Admix |
| **T.16636** | Cavimor |  |  |  | CATIE, Costa-Rica | Kent | HiPlex5 |
| **T.16651** | Cavimor |  |  |  | CATIE, Costa-Rica | Kent | Admix |
| **T.16742** | Mundo Novo | Brasil | Minas Gerais | Yes | CATIE, Costa-Rica | Admix | Admix |
| **T.16762** | Mundo Novo | Brasil | Minas Gerais | Yes | CATIE, Costa-Rica | Admix | Admix |
| **T.16764** | Catuai | Brasil | Minas Gerais | Yes | CATIE, Costa-Rica | Admix | Admix |
| **T.19878** | Arabusta |  |  |  | CATIE, Costa-Rica | Admix | Admix |
| **T.19943** | Arabusta |  |  |  | CATIE, Costa-Rica | Admix | Admix |
| **T.03617** | Blue Mountain |  |  |  | CATIE, Costa-Rica | Admix | Admix |
| **S15_2** | *C. canephora* | Guinea |  |  | IRD, France | N/A | N/A |
| **SBIL4** | *C. canephora var. maclaudi* |  |  |  | IRD, France | N/A | N/A |
| **Ku041** | Red_bourbon |  |  |  | HARC, Hawaii, USA | BBN | N/A |
| **Mw235** | Pink_bourbon |  |  |  | HARC, Hawaii, USA | BBN | N/A |
| **Mw264** | Bourbon Select PR_6791 |  |  |  | HARC, Hawaii, USA | BBN | N/A |
| **Ku117** | Typica_Guatemala |  |  |  | HARC, Hawaii, USA | Typ | N/A |
| **Ku118** | Typica_Guatemala |  |  |  | HARC, Hawaii, USA | Typ | N/A |
| **Ku132** | Typica_San_Ramon |  |  |  | HARC, Hawaii, USA | Typ | N/A |
| **Ku214** | Jamaica Blue_Mountain |  |  |  | HARC, Hawaii, USA | Typ | N/A |
| **Ku215** | Jamaica Blue_Mountain |  |  |  | HARC, Hawaii, USA | Typ | N/A |
| **Ku085** | 8667 |  |  |  | HARC, Hawaii, USA | CR | N/A |
| **Ku086** | 8667 |  |  |  | HARC, Hawaii, USA | CR | N/A |
| **Ku143** | Kents |  |  |  | HARC, Hawaii, USA | TK | N/A |
| **T.08667** | CR95 |  |  |  | CATIE, Costa-Rica | CR | N/A |
| **T.04271** | Kents_198 |  |  |  | CATIE, Costa-Rica | Admix | N/A |
| **T19844** | Hibrido_038 |  |  |  | CATIE, Costa-Rica | Kent | N/A |
| **T.05296** | Sarchimor |  |  |  | CATIE, Costa-Rica | Admix | N/A |
