# Supplementary material for: Haitian coffee agroforestry systems harbor complex arabica variety mixtures and under-recognized genetic diversity
Source: PLoS One. 2024 Apr 16;19(4):e0299493. doi: 10.1371/journal.pone.0299493 (PMC11020479; doi:10.1371/journal.pone.0299493)
Supplement: S3 Table — (DOCX) [file pone.0299493.s003.docx]

**Table S3. Haitian *Coffea arabica* diversity statistics calculated on SNP genotyping data for the sampled departments of Nord (N) and Grande-Anse (G), and both combined (N+G)**: sample size, observed heterozygosity (***H_o_***), expected heterozygosity (=gene diversity, ***H_e_*** ), Fixation index (as ***F_IS_***) and percent marker polymorphism (% P). Values calculated from reference *C. arabica* individuals (Arabica ref.) are included as a comparison. Where applicable, data is presented as Mean ± SD.

| **Department** | **Nb. of samples** | ***H_o_*** | | | ***H_e_*** | | | ***F_IS_*** | | | **% P** |
| --- | --- | --- | --- | --- | --- | --- | --- | --- | --- | --- | --- |
| **N** | 300 | 0.072 | ± | 0.01 | 0.332 | ± | 0.02 | 0.761 | ± | 0.03 | 0.908 |
| **G** | 301 | 0.093 | ± | 0.01 | 0.309 | ± | 0.02 | 0.666 | ± | 0.04 | 0.885 |
| **Overall (N+G samples)** | 601 | 0.083 | ± | 0.01 | 0.325 | ± | 0.02 | 0.724 | ± | 0.03 | 0.908 |
| **Arabica ref.** | 110 | 0.140 | ± | 0.01 | 0.373 | ± | 0.01 | 0.620 | ± | 0.03 | 0.989 |
