# Supplementary material for: Haitian coffee agroforestry systems harbor complex arabica variety mixtures and under-recognized genetic diversity
Source: PLoS One. 2024 Apr 16;19(4):e0299493. doi: 10.1371/journal.pone.0299493 (PMC11020479; doi:10.1371/journal.pone.0299493)
Supplement: S4 Table — (DOCX) [file pone.0299493.s004.docx]

**Table S4. Haitian *Coffea arabica* diversity statistics calculated on SNP genotyping data for the sampled municipalities (*communes*) in the Nord (N) and Grande-Anse (GA) departments**: sample size (N), observed heterozygosity (***H_o_***), expected heterozygosity (=gene diversity, ***H_e_*** ), Fixation index (as ***F_IS_***) and percent marker polymorphism (% P). Values calculated from reference *C. arabica* individuals (Arabica ref.) are included as a comparison. “GRN” = Grande Rivière du Nord. Where applicable, data is presented as Mean ± SD.

| ***Commune* (municipality)** | **Nb. of samples** | ***H_o_*** | | | ***H_e_*** | | | ***F_IS_*** | | | **% P** |
| --- | --- | --- | --- | --- | --- | --- | --- | --- | --- | --- | --- |
| **N-DONDON** | 210 | 0.082 | ± | 0.01 | 0.338 | ± | 0.02 | 0.740 | ± | 0.03 | 0.908 |
| **N-GRN** | 21 | 0.067 | ± | 0.01 | 0.311 | ± | 0.02 | 0.784 | ± | 0.03 | 0.793 |
| **N-BAHON** | 69 | 0.043 | ± | 0.02 | 0.161 | ± | 0.01 | 0.757 | ± | 0.04 | 0.793 |
| **G-BEAUMONT** | 148 | 0.081 | ± | 0.02 | 0.264 | ± | 0.02 | 0.705 | ± | 0.04 | 0.828 |
| **G-PESTEL** | 153 | 0.105 | ± | 0.01 | 0.337 | ± | 0.02 | 0.632 | ± | 0.04 | 0.874 |
| **Arabica ref.** | 110 | 0.140 | ± | 0.01 | 0.373 | ± | 0.01 | 0.620 | ± | 0.03 | 0.989 |
