# Supplementary material for: Haitian coffee agroforestry systems harbor complex arabica variety mixtures and under-recognized genetic diversity
Source: PLoS One. 2024 Apr 16;19(4):e0299493. doi: 10.1371/journal.pone.0299493 (PMC11020479; doi:10.1371/journal.pone.0299493)
Supplement: S5 Table — (DOCX) [file pone.0299493.s005.docx]

**Table S5. Haitian *Coffea arabica* diversity statistics for the sampled farms in the Nord (N) and Grande-Anse (G) departments, based on SNP genotyping data**: sample size (N), observed heterozygosity (***H_o_***), expected heterozygosity (=gene diversity, ***H_e_*** ), Fixation index (as ***F_IS_***) and percent marker polymorphism (% P). Values calculated from reference *C. arabica* individuals (Arabica ref.) are included as a comparison. Where applicable, data is presented as Mean ± SD.

| **Farm** | **Nb. of samples** | ***H_o_*** | | | ***H_e_*** | | | ***F_IS_*** | | | **% P** |
| --- | --- | --- | --- | --- | --- | --- | --- | --- | --- | --- | --- |
| **N01** | 24 | 0.045 | ± | 0.02 | 0.139 | ± | 0.01 | 0.678 | ± | 0.05 | 0.759 |
| **N02** | 25 | 0.043 | ± | 0.01 | 0.294 | ± | 0.02 | 0.804 | ± | 0.05 | 0.828 |
| **N03** | 24 | 0.075 | ± | 0.02 | 0.272 | ± | 0.02 | 0.713 | ± | 0.04 | 0.690 |
| **N04** | 26 | 0.096 | ± | 0.01 | 0.237 | ± | 0.02 | 0.584 | ± | 0.04 | 0.851 |
| **N05** | 19 | 0.099 | ± | 0.02 | 0.335 | ± | 0.02 | 0.677 | ± | 0.04 | 0.885 |
| **N06** | 21 | 0.102 | ± | 0.01 | 0.305 | ± | 0.02 | 0.618 | ± | 0.04 | 0.839 |
| **N07** | 24 | 0.098 | ± | 0.01 | 0.343 | ± | 0.02 | 0.693 | ± | 0.03 | 0.759 |
| **N08** | 24 | 0.083 | ± | 0.01 | 0.292 | ± | 0.02 | 0.667 | ± | 0.04 | 0.839 |
| **N09** | 23 | 0.095 | ± | 0.01 | 0.292 | ± | 0.02 | 0.615 | ± | 0.04 | 0.770 |
| **N10** | 21 | 0.067 | ± | 0.01 | 0.311 | ± | 0.02 | 0.784 | ± | 0.03 | 0.793 |
| **N11** | 20 | 0.062 | ± | 0.01 | 0.309 | ± | 0.02 | 0.747 | ± | 0.04 | 0.793 |
| **N12** | 9 | 0.036 | ± | 0.02 | 0.022 | ± | 0.01 | -0.569 | ± | 0.09 | 0.046 |
| **N13** | 20 | 0.036 | ± | 0.02 | 0.029 | ± | 0.01 | -0.188 | ± | 0.10 | 0.069 |
| **N14** | 20 | 0.036 | ± | 0.02 | 0.026 | ± | 0.01 | -0.209 | ± | 0.10 | 0.069 |
| **G05** | 24 | 0.087 | ± | 0.01 | 0.336 | ± | 0.02 | 0.727 | ± | 0.04 | 0.805 |
| **G06** | 20 | 0.075 | ± | 0.02 | 0.186 | ± | 0.02 | 0.581 | ± | 0.05 | 0.759 |
| **G07** | 22 | 0.138 | ± | 0.02 | 0.265 | ± | 0.02 | 0.532 | ± | 0.04 | 0.793 |
| **G08** | 20 | 0.046 | ± | 0.02 | 0.198 | ± | 0.02 | 0.746 | ± | 0.05 | 0.632 |
| **G09** | 22 | 0.103 | ± | 0.01 | 0.319 | ± | 0.02 | 0.633 | ± | 0.04 | 0.782 |
| **G10** | 20 | 0.073 | ± | 0.02 | 0.101 | ± | 0.01 | 0.399 | ± | 0.05 | 0.678 |
| **G11** | 20 | 0.032 | ± | 0.02 | 0.217 | ± | 0.02 | 0.852 | ± | 0.05 | 0.724 |
| **G12** | 26 | 0.153 | ± | 0.02 | 0.331 | ± | 0.02 | 0.552 | ± | 0.04 | 0.828 |
| **G13** | 20 | 0.034 | ± | 0.02 | 0.017 | ± | 0.01 | -1.000 | ± | 0.00 | 0.034 |
| **G14** | 23 | 0.036 | ± | 0.02 | 0.055 | ± | 0.01 | 0.774 | ± | 0.06 | 0.506 |
| **G15** | 21 | 0.095 | ± | 0.01 | 0.319 | ± | 0.02 | 0.687 | ± | 0.04 | 0.805 |
| **G16** | 20 | 0.113 | ± | 0.01 | 0.283 | ± | 0.02 | 0.542 | ± | 0.04 | 0.747 |
| **G17** | 21 | 0.133 | ± | 0.02 | 0.272 | ± | 0.02 | 0.510 | ± | 0.04 | 0.759 |
| **G18** | 22 | 0.159 | ± | 0.01 | 0.272 | ± | 0.02 | 0.377 | ± | 0.03 | 0.839 |
| **Arabica ref.** | 110 | 0.140 | ± | 0.01 | 0.373 | ± | 0.01 | 0.620 | ± | 0.03 | 0.989 |
