# Supplementary material for: Haitian coffee agroforestry systems harbor complex arabica variety mixtures and under-recognized genetic diversity
Source: PLoS One. 2024 Apr 16;19(4):e0299493. doi: 10.1371/journal.pone.0299493 (PMC11020479; doi:10.1371/journal.pone.0299493)
Supplement: S6 Table — (DOCX) [file pone.0299493.s006.docx]

**Table S6. Pairwise *F_ST_* values between Haitian *Coffea arabica* sampled in municipalities** (*communes,* M) of two departments (D), calculated from KASP SNP genotyping data. “GRN” refers to Grande Rivière du Nord. p-values above diagonal, based on 999 permutations, are as follows: * = p≤0.05, **= p≤0.01, ***= p≤0.001

| **D** |  | **Grande-Anse** | | **Nord** | | |
| --- | --- | --- | --- | --- | --- | --- |
|  | **M** | **Beaumont** | **Pestel** | **Dondon** | **GRN** | **Bahon** |
| **Grande-Anse** | **Beaumont** |  |  | ***** | ***** | ***** |
|  | **Pestel** | 0.045 |  | ***** | ***** | ***** |
| **Nord** | **Dondon** | 0.118 | 0.028 |  | *** | ***** |
|  | **GRN** | 0.204 | 0.063 | 0.020 |  | ***** |
|  | **Bahon** | 0.043 | 0.146 | 0.225 | 0.388 |  |
