# Supplementary material for: Haitian coffee agroforestry systems harbor complex arabica variety mixtures and under-recognized genetic diversity
Source: PLoS One. 2024 Apr 16;19(4):e0299493. doi: 10.1371/journal.pone.0299493 (PMC11020479; doi:10.1371/journal.pone.0299493)
Supplement: S7 Table — (DOCX) [file pone.0299493.s007.docx]

**Table S7. Pairwise *F_ST_* values between Haitian *Coffea arabica* sampled in farms** (F) from five municipalities (M) and two departments (D), calculated from KASP SNP genotyping data. “GRN” refers to Grande Rivière du Nord. p-values above diagonal, based on 999 permutations, are as follows: * = p≤0.05, **= p≤0.01, ***= p≤0.001, ns= not significant.
