# Supplementary material for: Haitian coffee agroforestry systems harbor complex arabica variety mixtures and under-recognized genetic diversity
Source: PLoS One. 2024 Apr 16;19(4):e0299493. doi: 10.1371/journal.pone.0299493 (PMC11020479; doi:10.1371/journal.pone.0299493)
Supplement: S8 Table — (DOCX) [file pone.0299493.s008.docx]

**Table S8. Correspondence between genetic groups defined on KASP SNP data or HiPlex haplotype data.** Genetic groups were determined for Haitian and reference samples of *Coffea arabica* based on an 80% threshold of membership in ancestral populations determined by population structure analyses at K=7 and K=6 for HiPlex and KASP genotyping, respectively. Data is presented as number of samples from any KASP group that is assigned to a HiPlex group, and percentage so that the sum of percentages for each KASP group amounts to 100%.

|  |  | **KASP SNP data** | | | | | | |
| --- | --- | --- | --- | --- | --- | --- | --- | --- |
|  | **Group** | **Typica-like (N=252)** | **CR95/ Catimor-like (N=72)** | **« Unlabeled » Haitian samples (N=51)** | **Bourbon-like (N=32)** | **Ethiopian-like (N=20)** | **Kent/I-60-like (N=8)** | **Admixed (N=249)** |
| **HiPlex haplotype data** | **HiPlex1 (N=241)** | **198 (78.6%)** | 9 (12.5%) | 6 (11.8%) | 6 (18.8%) | - | 0.0 | 22 (8.8%) |
|  | **HiPlex2 (N=71)** | 9 (3.6%) | **50 (69.4%)** | 1 (2.0%) | - | - | **2 (25.0%)** | 9 (3.6%) |
|  | **HiPlex3 (N=51)** | 4 (1.6%) | 1 (1.4%) | **31 (60.8%)** | 1 (3.1%) | - | 0.0 | 14 (5.6%) |
|  | **HiPlex4 (N=30)** | 1 (0.4%) | - | 2 (3.9%) | 1 (3.1%) | - | 0.0 | 26 (10.4%) |
|  | **HiPlex5 (N=26)** | 5 (2.0%) | 1 (1.4%) | - | **15 (46.9%)** | - | 0.0 | 5 (2.0%) |
|  | **HiPlex6 (N=17)** | - | - | - | - | **15 (75.0%)** | 0.0 | 2 (0.8%) |
|  | **HiPlex7 (N=4)** | - | 2 (2. 8%) | - | - | - | **2 (25.0%)** | 0.0 |
|  | **Admixed (N=244)** | 35 (13. 9%) | 9 (12.5%) | 11 (21.6%) | 9 (28.1%) | 5 (25.0%) | **4 (50.0%)** | **171 (68.7%)** |
