# Supplementary material for: Haitian coffee agroforestry systems harbor complex arabica variety mixtures and under-recognized genetic diversity
Source: PLoS One. 2024 Apr 16;19(4):e0299493. doi: 10.1371/journal.pone.0299493 (PMC11020479; doi:10.1371/journal.pone.0299493)
Supplement: S1 Fig — (DOCX) [file pone.0299493.s009.docx]

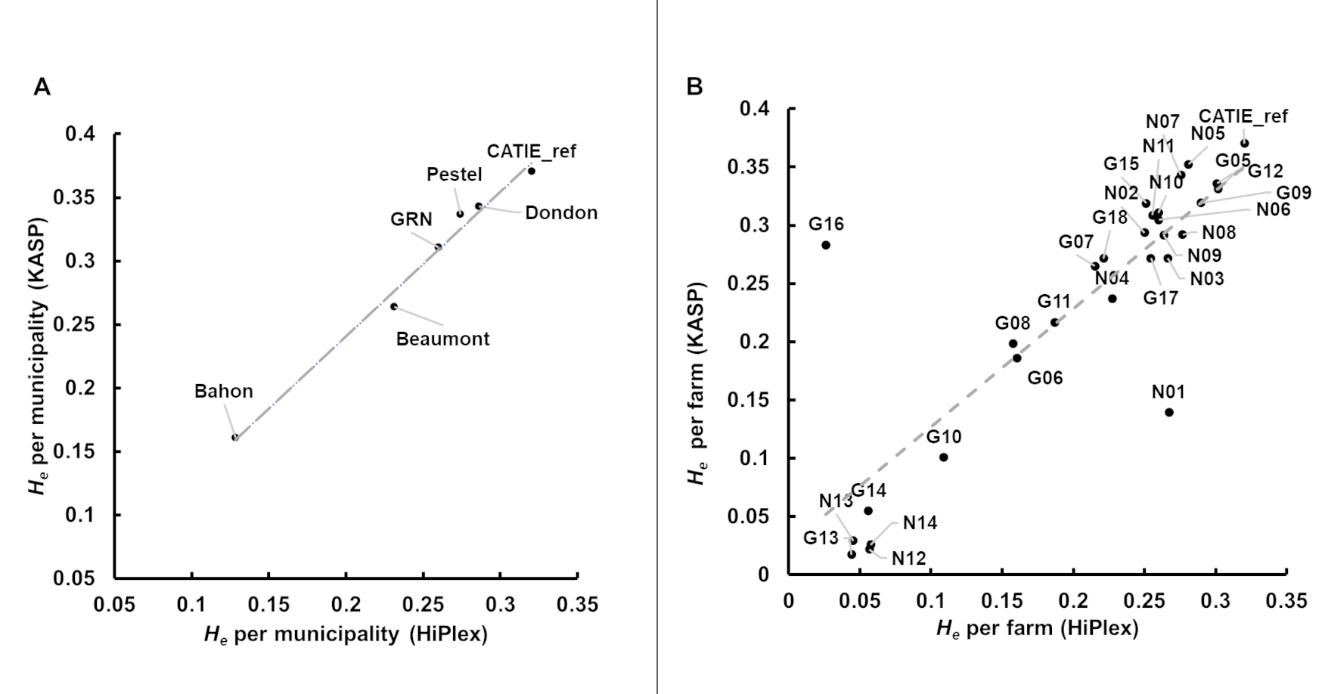


**Figure S1. Expected heterozygosity (*H_e_*) calculated on HiPlex haplotype genotyping *versus* KASP SNP genotyping.** **A.** Plot of HiPlex- *vs.* KASP-based *H_e_* values for sampled municipalities (*communes*) in Haiti (« GRN » refers to Grande Rivière du Nord, trend line in grey: y=1.13x+0.01, R^2^=0.99). **B.** Plot of HiPlex- *vs.* KASP-based *H_e_* values for sampled farms in the Nord (N) and Grande-Anse (G) departments (trend line in grey: y=1.01x+0.03, R^2^=0.71). In both plots, *H_e_* value calculated on reference samples from the CATIE international collection (N=96) are included for comparison.
