# Supplementary material for: Haitian coffee agroforestry systems harbor complex arabica variety mixtures and under-recognized genetic diversity
Source: PLoS One. 2024 Apr 16;19(4):e0299493. doi: 10.1371/journal.pone.0299493 (PMC11020479; doi:10.1371/journal.pone.0299493)
Supplement: S2 Fig — (DOCX) [file pone.0299493.s010.docx]

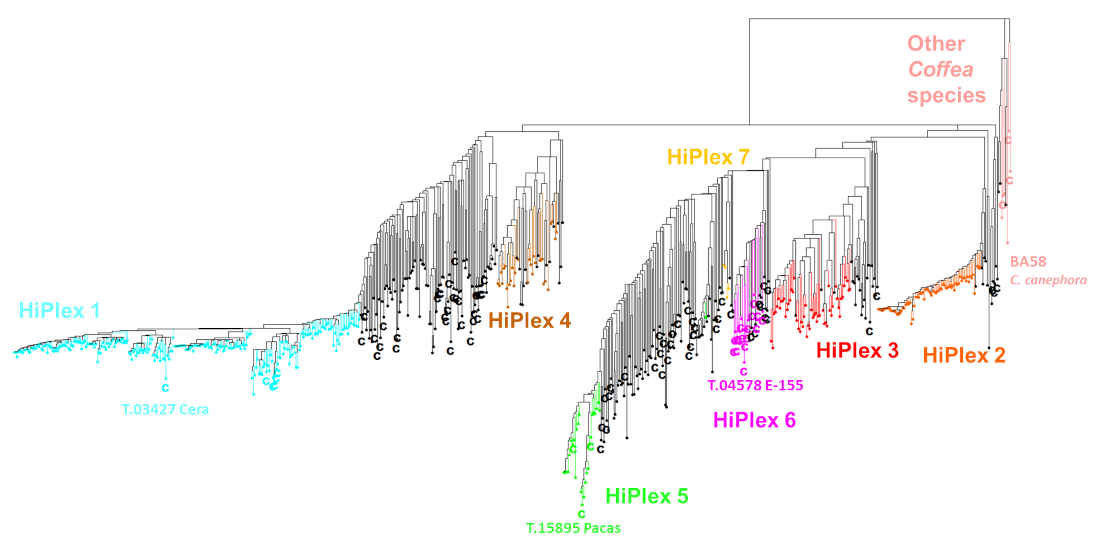


**Figure S2. Unweighted neighbor-joining dendrogram of Haitian coffee samples and reference samples from collections (“C”) based on SNP data from HiPlex haplotype data**. Individuals were colored according to a 80% threshold of membership in ancestral populations identified by population structure analysis (K=7). All samples are *Coffea arabica* except where indicated at the root of the dendrogram. Select reference individuals are labeled for illustrative purposes.
