# Supplementary material for: Haitian coffee agroforestry systems harbor complex arabica variety mixtures and under-recognized genetic diversity
Source: PLoS One. 2024 Apr 16;19(4):e0299493. doi: 10.1371/journal.pone.0299493 (PMC11020479; doi:10.1371/journal.pone.0299493)
Supplement: S3 Fig — Supplementary Note: HiPlex read processing bioinformatics pipeline. (DOCX) [file pone.0299493.s011.docx]

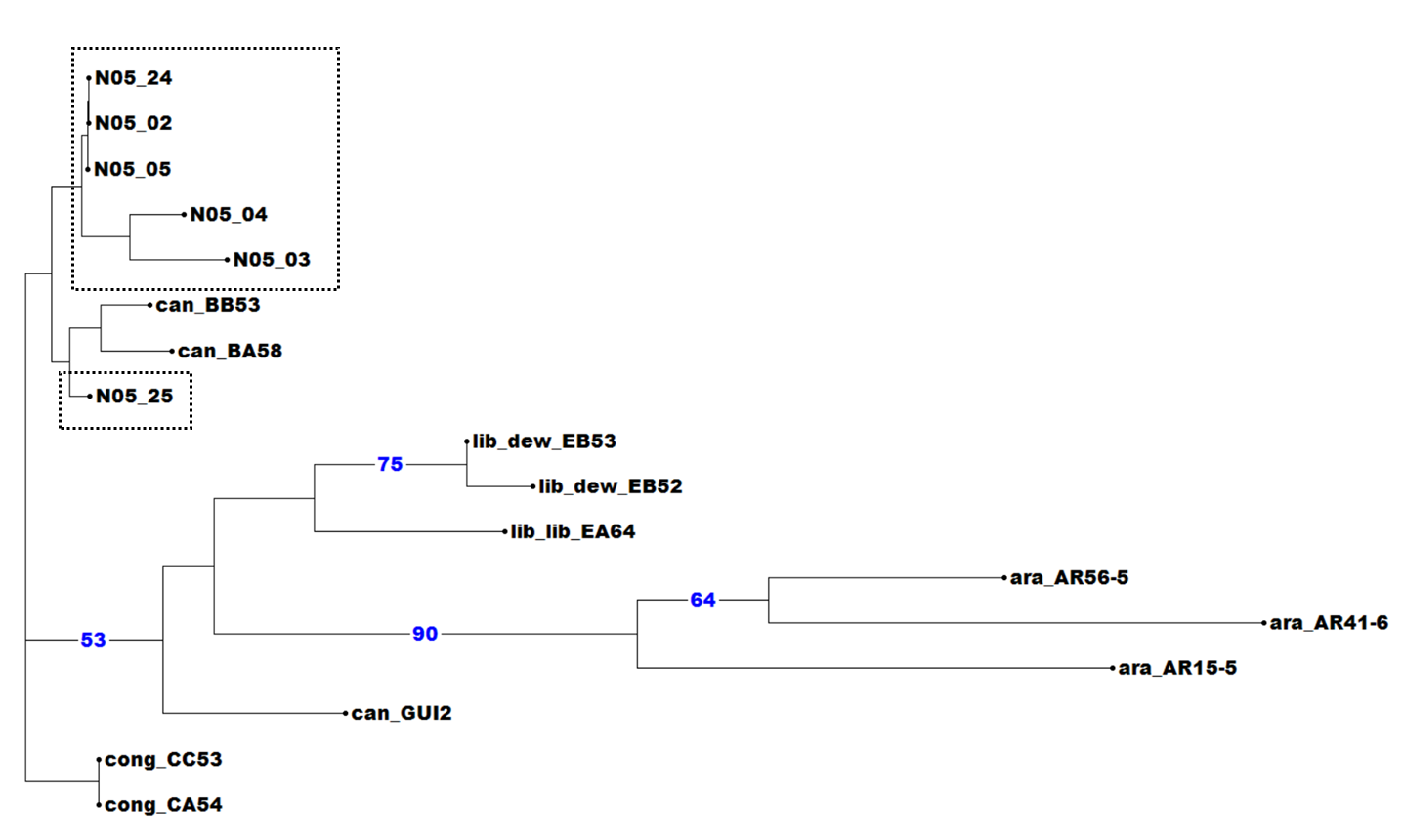


**Figure S3. Unweighted neighbor-joining dendrogram of Haitian Robusta coffee trees and reference samples.** Haitian samples are in dotted frames**.** The tree was built from a simple-matching distance matrix calculated from KASP SNP genotyping data. Prefixes in reference sample names refer to their species: can = *Coffea canephora*, lib_lib = *C. liberica liberica*, lib_dew = *C. liberica dewevrei*, ara = *C. arabica* and cong = *C. congensis*. Bootstrap values (1000 repetitions) are shown when >50.

# Supplementary Note: HiPlex read processing bioinformatics pipeline

HiPlex reads were mapped onto each of the two subgenomes of the *C. arabica* reference genome sequence v0.6 of the accession ET-39 (Salojärvi et al., in press) using the BWA-MEM algorithm in BWA v0.7.17 (Li, 2013) with default settings. To correctly classify reads to the respective *C. arabica* subgenomes, a genome read categorization approach was applied using a customized script by Bawin (2020), available on GitLab (https://gitlab.com/ybawin/sequence-data-processing-tetraploids). After filtering out ambiguously mapped reads, new ‘categorized’ read mapping (BAM) files were created with only high quality (subgenome-specific) mapped reads. These BAM files were indexed with Samtools v1.10 (Li et al., 2009), and read groups were added with Picard v2.18.25 (Picard Toolkit, 2019). Single nucleotide polymorphisms (SNPs) were called with GATK (Genome Analysis Toolkit) Unified Genotyper v3.7.0 (McKenna et al., 2010). SNPs were filtered using the following parameters: min-meanDP 30, mac 4, and minQ 20, and multi-allelic SNPs were removed with GATK. The remaining SNPs were then subjected to further filtering with the following parameters: minDP 10, minGQ 30, minQ 30, min-alleles 2, max-alleles 2, and maf 0.05 using VCFtools v0.1.16 (Danecek et al., 2011). Read-backed haplotyping was conducted based on SNPs in the HiPlex read data using module *SMAP haplotype-sites* of the SMAP software package v4.2.0 (Schaumont et al., 2022, https://gitlab.ilvo.be/genomics/smap) with mapping_orientation ignore, partial exclude, no_indels, min_read_count 10, min_distinct_haplotypes 2, min_haplotype_frequency 5, discrete_calls dosage, frequency_interval_bounds 10 10 90 90, dosage_filter 2, and completeness 30. Genotype tables were then filtered to exclude loci with missing data in >30% of Arabica samples, then individuals of all species with >30% missing genotype data.

**Pipeline References**

Broad Institute, GitHub repository, 2019. Picard Toolkit.

Danecek, P., Auton, A., Abecasis, G., Albers, C.A., Banks, E., DePristo, M.A., Handsaker, R.E., Lunter, G., Marth, G.T., Sherry, S.T., McVean, G., Durbin, R., 1000 Genomes Project Analysis Group, 2011. The variant call format and VCFtools. Bioinformatics 27, 2156–2158. https://doi.org/10.1093/bioinformatics/btr330

Li, H., 2013. Aligning sequence reads, clone sequences and assembly contigs with BWA-MEM.

Li, H., Handsaker, B., Wysoker, A., Fennell, T., Ruan, J., Homer, N., Marth, G., Abecasis, G., Durbin, R., 1000 Genome Project Data Processing Subgroup, 2009. The Sequence Alignment/Map format and SAMtools. Bioinformatics 25, 2078–2079. https://doi.org/10.1093/bioinformatics/btp352

McKenna, A., Hanna, M., Banks, E., Sivachenko, A., Cibulskis, K., Kernytsky, A., Garimella, K., Altshuler, D., Gabriel, S., Daly, M., DePristo, M.A., 2010. The Genome Analysis Toolkit: A MapReduce framework for analyzing next-generation DNA sequencing data. Genome Res. 20, 1297–1303. https://doi.org/10.1101/gr.107524.110

Salojärvi, J., Rambani, A., Yu, Z., Guyot, R., Strickler, S., Lepelley, M., Wang, C., Rajaraman, S., Rastas, P., Zheng, C., Munoz, D.S., Meidanis, J., Paschoal, A.R., Bawin, Y., Krabbenhoft, T., Wang, Z.Q., Fleck, S., Aussel, R., Bellanger, L., Charpagne, A., Fournier, C., Kassam, M., Lefebvre, G., Metairon, S., Moine, D., Rigoreau, M., Stolte, J., Hamon, P., Couturon, E., Tranchant-Dubreuil, C., Mukherjee, M., Lan, T., Engelhardt, J., Stadler, P., DeLemos, S.C., Suzuki, S.I., Sumirat, U., ChingMan, W., Dauchot, N., Orozco-Arias, S., Garavito, A., Kiwuka, C., Musoli, P., Nalukenge, A., Guichoux, E., Reinout, H., Smit, M., Carretero-Paulet, L., Filho, O.G., Braghini, M.T., Padilha, L., Sera, G.H., Ruttink, T., Henry, R., Marraccini, P., Peer, Y.V. de, Andrade, A., Domingues, D., Giuliano, G., Mueller, L., Pereira, L.F., Plaisance, S., Poncet, V., Rombauts, S., Sankoff, D., Albert, V.A., Crouzillat, D., deKochko, A., Descombes, P., (in press). The genome and population genomics of allopolyploid Coffea arabica reveal the diversification history of modern coffee cultivars. Nature Genetics.

Schaumont, D., Veeckman, E., Van Der Jeugt, F., Haegeman, A., Van Glabeke, S., Bawin, Y., Lukasiewicz, J., Blugeon, S., Barre, P., Leyva-Pérez, M.D.L.O., Byrne, S., Dawyndt, P., Ruttink, T., 2022. Stack Mapping Anchor Points (SMAP): a versatile suite of tools for read-backed haplotyping (preprint). Genomics. https://doi.org/10.1101/2022.03.10.483555
